# Identification and Genetic Characterization of Conjugative Plasmids Encoding Coresistance to Ciprofloxacin and Cephalosporin in Foodborne *Vibrio* spp.

**DOI:** 10.1128/spectrum.01032-23

**Published:** 2023-07-03

**Authors:** Yating Xu, Zhiwei Zheng, Lianwei Ye, Edward Wai-Chi Chan, Sheng Chen

**Affiliations:** a Department of Infectious Diseases and Public Health, Jockey Club College of Veterinary Medicine and Life Sciences, City University of Hong Kong, Kowloon, Hong Kong; b City University of Hong Kong Chengdu Research Institute, Chengdu, People’s Republic of China; c Shenzhen Key Laboratory for Food Biological Safety Control, Food Safety and Technology Research Centre, The Hong Kong PolyU Shenzhen Research Institute, Shenzhen, People’s Republic of China; d State Key Laboratory of Chirosciences and the Department of Food Science and Nutrition, The Hong Kong Polytechnic University, Kowloon, Hong Kong; Yale University

**Keywords:** foodborne *Vibrio* spp., antimicrobial resistance, plasmid-mediated quinolone resistance, *qnrS* gene, pAQU-like plasmid

## Abstract

Plasmid-mediated quinolone resistance (PMQR) determinants, such as *qnrVC* genes, have been widely reported in *Vibrio* spp. while other types of PMQR genes were rarely reported in these bacteria. This study characterized the phenotypic and genotypic features of foodborne *Vibrio* spp. carrying *qnrS*, a key PMQR gene in *Enterobacteriaceae*. Among a total of 1,811 foodborne *Vibrio* isolates tested, 34 (1.88%) were found to harbor the *qnrS* gene. The allele *qnrS2* was the most prevalent, but coexistence with other *qnr* alleles was common. Missense mutations in the quinolone resistance-determining region (QRDR) of the *gyrA* and *parC* genes were only found in 11 of the 34 *qnrS*-bearing isolates. Antimicrobial susceptibility tests showed that all 34 *qnrS*-bearing isolates were resistant to ampicillin and that a high percentage also exhibited resistance to cefotaxime, ceftriaxone, and trimethoprim-sulfamethoxazole. Genetic analysis showed that these phenotypes were attributed to a diverse range of resistance elements that the *qnrS*-bearing isolates harbored. The *qnrS2* gene could be found in both the chromosome and plasmids; the plasmid-borne *qnrS2* genes could be found on both conjugative and nonconjugative plasmids. pAQU-type *qnrS2*-bearing conjugative plasmids were able to mediate expression of phenotypic resistance to both ciprofloxacin and cephalosporins. Transmission of this plasmid among *Vibrio* spp. would speed up the emergence of multidrug-resistant (MDR) pathogens that are resistant to the most important antibiotics used in treatment of *Vibrio* infections, suggesting that close monitoring of emergence and dissemination of MDR *Vibrio* spp. in both food samples and clinical settings is necessary.

**IMPORTANCE** *Vibrio* spp. used to be very susceptible to antibiotics. However, resistance to clinically important antibiotics, such as cephalosporins and fluoroquinolones, among clinically isolated *Vibrio* strains is increasingly common. In this study, we found that plasmid-mediated quinolone resistance (PMQR) genes, such as *qnrS*, that have not been previously reported in *Vibrio* spp. can now be detected in food isolates. The *qnrS2* gene alone could mediate expression of ciprofloxacin resistance in *Vibrio* spp.; importantly, this gene could be found in both the chromosome and plasmids. The plasmids that harbor the *qnrS2* gene could be both conjugative and nonconjugative, among which the pAQU-type *qnrS2*-bearing conjugative plasmids were able to mediate expression of resistance to both ciprofloxacin and cephalosporins. Transmission of this plasmid among *Vibrio* spp. would accelerate the emergence of multidrug-resistant pathogens.

## INTRODUCTION

*Vibrio* spp. are water- and seafood-borne bacterial pathogens that can cause large-scale outbreaks of gastrointestinal infections (1). Quinolones are among the most important antibacterial agents used in the clinical treatment of bacterial infections and in the field of veterinary medicine (2). Fluoroquinolones (FQs), which are second-generation quinolones, exhibit noticeably enhanced activity against a range of Gram-negative and Gram-positive bacteria (3, 4) and are widely used synthetic broad-spectrum antibiotics that are also recommended for the treatment of *Vibrio* infections in humans (4). However, recent studies identified a range of underlying mechanisms of resistance in fluoroquinolone-resistant *Vibrio* spp.; these include chromosomal mutations in the quinolone resistance-determining region (QRDR) of the genes encoding DNA gyrase (*gyrA* and *gyrB*) and DNA topoisomerase IV (*parC* and *parE*). It was found that such mutational changes were often associated with changes in the expression levels of various outer membrane proteins or efflux pumps (5, 6). In addition, plasmid-mediated quinolone resistance (PMQR) determinants, which encode a range of resistance-conferring proteins, including the Qnr proteins, the aminoglycoside acetyltransferase AAC(6′)-Ib-cr, and the efflux pumps QepA and OqxAB, have been reported to confer low-level resistance to fluoroquinolones (7, 8). Unlike the target site mutations, which can be passed onto future generations, the plasmid-borne PMQR genes can not only enhance the ability of bacteria to survive against the antimicrobial effects of antibiotics but also accelerate the dissemination of resistance genes among a diverse range of bacterial species through horizontal transfer of the resistance-encoding plasmids (9, 10).

The PMQR gene *qnrS* was first discovered in 2006. The gene, which was recovered from an S. flexneri 2b strain isolated in Japan, was found to be located in a 47-kb conjugative plasmid, pAH0376 (11, 12). To date, nine *qnrS* alleles have been documented; these genes were recovered from various types of microorganisms and the environment; more recently, six other alleles (*qnrS10* to *qnrS15*) have been identified, and their sequences have been deposited into GenBank (13). Previous studies showed that *qnrS* genes could be located in both the chromosome and plasmids. The mobilization and dissemination of *qnrS* alleles are known to be mediated by insertion sequences (IS), including IS*2*, IS*26*, and IS*Ec1* (14, 15). In addition, *qnrS* has also been discovered in the vicinity of Tn*3*-like structures carrying *bla*_TEM-1_ (16). Cephalosporins are a large group of related β-lactam antimicrobial drugs. The third-generation class of cephalosporins, such as cefotaxime and ceftriaxone, are broad-spectrum antimicrobial agents most commonly used in the management and treatment of infections caused by both Gram-negative and Gram-positive pathogens. Bacterial resistance to third-generation cephalosporins is attributed to the production of β-lactamases, including extended-spectrum β-lactamases (ESBLs), AmpCs, and carbapenemases (17, 18). The spread of genes encoding extended-spectrum β-lactamase among diverse pathogens poses a major threat to the treatment of bacterial infections.

In the present study, we investigated the carriage of PMQR genes among foodborne *Vibrio* spp., particularly *qnrS*, which has been shown to mediate expression of phenotypic resistance to ciprofloxacin in other bacterial pathogens, such as Salmonella (19). We also analyzed the genetic background of the *qnrS* gene and the transferability of the plasmids harboring this gene. Our findings in this work not only identified and characterized PMQR genes, such as *qnrS* in *Vibrio* spp., for the first time but also identified conjugative plasmids that encode coresistance to ciprofloxacin and cephalosporins, which provides important insight into the rapid evolution of foodborne *Vibrio* spp. that exhibit resistance to clinically important antibiotics and warrants further monitoring and investigation.

## RESULTS

**FIG 1:**
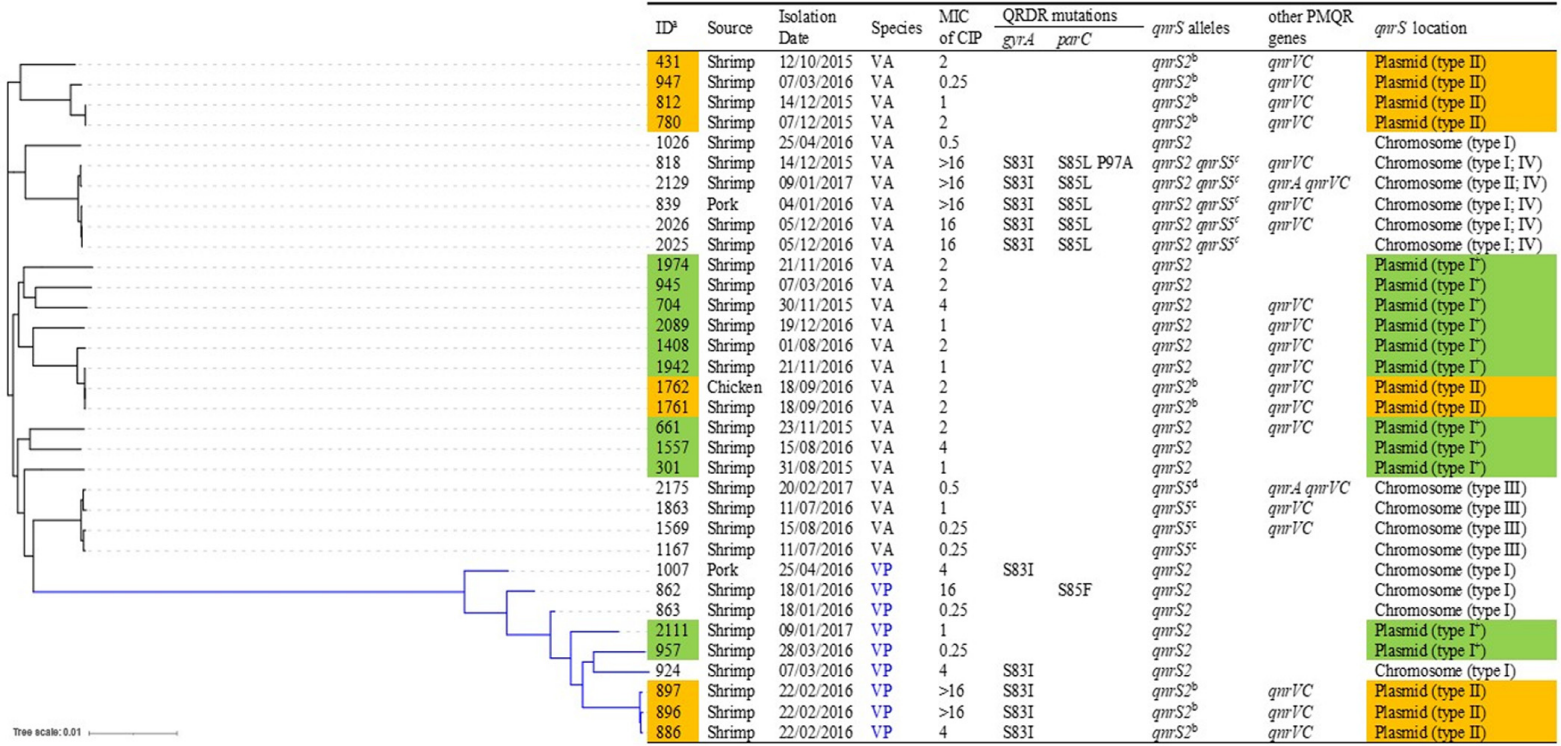
Maximum likelihood phylogenetic tree and strain information of *qnrS*-carrying *Vibrio* spp. included in this study based on SNPs of core genomes. a, Abbreviations: ID, identification; CIP, ciprofloxacin; VA, Vibrio alginolyticus; VP, Vibrio parahaemolyticus. b, D218E substitution in QnrS2. c, R5N, R35C, A105V, H106N, T123N, V192I, V214I, and F216Y substitutions in QnrS5. d, R5N, R35C, V61A, H106N, T123N, R161L, V192I, V214I, and F216Y substitutions in QnrS5; +, conjugative plasmid.

Among a total of 1,811 *Vibrio* species strains isolated, 34 (1.88%) were found to be positive for the *qnrS* genes by PCR tests (Fig. 1), including 25 Vibrio alginolyticus and 9 Vibrio parahaemolyticus. Of the 34 *qnrS-*carrying *Vibrio* species isolates, 91.18% (31/34) were isolated from shrimp. Only two were from pork, and one was isolated from chicken. Most of these *qnrS*-positive strains were isolated in the year 2016 (24/34, 70.59%), followed by the year 2015 (7/34, 20.59%). Only three strains were identified in 2017. Whole-genome sequencing was performed on these 34 strains, with results confirming the presence of the *qnrS* gene. Multilocus sequence typing (MLST) analysis of nine V. parahaemolyticus strains indicated that they belonged to four sequence types (STs), including ST2264 (strains 886, 896, and 897), ST1042 (strain 924), ST1043 (strain 1007), and ST2257 (strain 2111). Sequence analysis showed that *qnrS2* was the most prevalent allele (25 out of 34, 73.53%), followed by *qnrS5* (4 out of 34, 11.76%). Interestingly, *qnrS2* and *qnrS5* were found to coexist in five isolates (14.71%). Nineteen isolates positive for the *qnrS* gene were also found to harbor the *qnrVC* gene. Notably, there were two isolates that carried three PMQR determinants, namely, *qnrS*, *qnrA*, and *qnrVC* (Fig. 1). DNA sequencing of the QRDR of the *gyrA* and *parC* genes of the 34 *qnrS*-positive *Vibrio* isolates revealed that point mutations leading to amino acid substitutions exist in 11 isolates. In the case of *gyrA*, only one type of amino acid change, namely, a change from serine to isoleucine in codon 83 of the GyrA protein, could be detected in 29.41% (10/34) of the isolates. Point mutations in *gyrA* at position 83 (Ser→Ile) and *parC* at position 85 (Ser→Leu) were only detected in four V. alginolyticus isolates (strains 839, 2025, 2026, and 2129). Additionally, a double mutation in *parC* (Ser85Leu and Pro97Ala) and a mutation in codon 83 of *gyrA* (Ser83Ile) were observed in V. alginolyticus strain 818. Moreover, a mutation in the *parC* gene that caused the Ser85Phe substitution was only detected in V. parahaemolyticus strain 862. However, no point mutations were observed in the genes *gyrB* and *parE*.

Antimicrobial susceptibility tests of the 34 *qnrS*-bearing isolates showed that all test strains were resistant to ampicillin and that a high percentage of these isolates exhibited resistance to cefotaxime, ceftriaxone, and trimethoprim-sulfamethoxazole (79.41%, 79.41%, and 82.35%, respectively). However, the rates of resistance to amoxicillin-clavulanic acid and chloramphenicol were 29.41% and 23.53%, respectively. All test strains remained susceptible to amikacin and meropenem and exhibited a high rate of susceptibility to gentamicin (33/34, 97.06%). The rates of resistance to three quinolone antibiotics, namely, nalidixic acid, ciprofloxacin, and ofloxacin, were 32.35%, 38.24%, and 23.53%, respectively. As much as 97.06% (33/34) of the test strains were regarded as multidrug resistant (MDR), exhibiting resistance to more than three types of antibiotics (Table 1). It should be noted that all 34 *qnrS*-bearing isolates exhibited reduced susceptibility to ciprofloxacin (MIC of ≥0.25 μg/mL).

**TABLE 1:** Antimicrobial susceptibility profiles of 34 foodborne *qnrS*-bearing *Vibrio* species isolates

Strain ID	MIC (μg/mL)^*a*^^,^^*b*^												
	AMP	CRO	CTX	MEM	AMC	AMK	GEN	TET	CHL	SXT	NAL	CIP	OFX
301	**>64**	0.12	0.06	0.0075	8	1	1	**16**	**32**	**>8**	8	1	1
431	**>64**	**>16**	**>16**	0.12	**32**	2	0.5	8	4	**>8**	8	2	1
661	**>64**	**>16**	**>16**	0.06	**32**	2	1	**16**	**32**	**>8**	8	2	1
704	**>64**	**>16**	**>16**	0.12	8	2	1	**32**	16	**>8**	4	**4**	1
780	**>64**	**>16**	**>16**	0.0075	4	2	1	2	4	**>8**	8	2	2
812	**>64**	**>16**	**>16**	0.0075	4	1	1	2	4	**>8**	4	1	2
818	**>64**	**>16**	**16**	0.0075	8	4	1	8	16	**>8**	**>128**	**>16**	**>16**
839	**>64**	0.06	0.12	0.0075	16	4	1	4	**>32**	**>8**	**>128**	**>16**	**>16**
862	**>64**	0.06	0.06	0.0075	8	4	2	**16**	**>32**	**>8**	**>128**	**16**	**>16**
863	**>64**	0.03	0.03	0.0075	8	4	1	0.25	0.5	1	2	0.25	0.25
886	**>64**	**>16**	**>16**	0.0075	4	4	1	4	8	**>8**	**>128**	**4**	4
896	**>64**	**>16**	**>16**	0.0075	4	4	1	4	8	**>8**	**>128**	**>16**	**8**
897	**>64**	**>16**	**>16**	0.0075	4	4	1	4	8	**>8**	**>128**	**>16**	**8**
924	**64**	0.03	0.06	0.0075	2	4	2	**16**	8	**>8**	**64**	**4**	4
945	**>64**	**>16**	**>16**	0.06	**32**	4	1	**16**	16	**>8**	4	2	0.5
947	**>64**	**>16**	**>16**	0.0075	4	2	1	4	4	**>8**	1	0.25	0.25
957	**>64**	**>16**	**>16**	0.0075	16	8	**32**	0.5	4	**>8**	8	0.5	1
1007	**>64**	0.03	0.06	0.0075	4	8	2	8	4	**>8**	16	**4**	2
1026	**>64**	0.06	0.25	0.0075	8	4	2	8	4	**>8**	**>128**	0.5	0.5
1167	**>64**	**>16**	**>16**	0.0075	8	2	2	2	0.5	0.25	4	0.25	0.5
1408	**>64**	**>16**	**>16**	0.06	4	2	2	**>32**	1	0.25	8	2	1
1557	**>64**	**>16**	**>16**	0.06	**32**	4	1	**32**	8	**>8**	8	**4**	2
1569	**>64**	**>16**	**>16**	0.06	**32**	8	1	8	4	**>8**	4	0.25	0.5
1761	**>64**	**>16**	**>16**	0.03	16	2	1	4	2	**>8**	2	2	0.5
1762	**>64**	**>16**	**>16**	0.03	16	2	1	4	2	<0.25	2	2	0.5
1863	**>64**	**>16**	**>16**	0.0075	16	2	2	**16**	**32**	**8**	4	1	0.5
1942	**>64**	**>16**	**16**	0.06	16	8	2	**32**	16	**>8**	2	1	0.5
1974	**>64**	**>16**	**>16**	0.015	16	2	0.5	**32**	**>32**	**>8**	2	2	2
2025	**>64**	**>16**	**16**	0.0075	**32**	1	1	**16**	1	**8**	**>128**	**16**	**16**
2026	**>64**	**16**	**16**	0.015	**32**	1	1	**16**	4	**8**	**>128**	**16**	**16**
2089	**>64**	**>16**	**>16**	0.12	**64**	4	1	**32**	**32**	**8**	2	1	0.25
2111	**>64**	**>16**	**>16**	0.25	16	4	2	**32**	**>32**	**8**	4	1	0.5
2129	**>64**	**>16**	**>16**	0.25	**32**	4	2	8	8	<0.25	**>128**	**>16**	**>16**
2175	**>64**	**>16**	**>16**	0.12	**64**	8	8	1	1	<0.25	8	0.5	1

a AMP, ampicillin; CRO, ceftriaxone; CTX, cefotaxime; MEM, meropenem; AMC, amoxicillin-clavulanic acid; AMK, amikacin; GEN, gentamicin; TET, tetracycline; CHL, chloramphenicol; SXT, trimethoprim-sulfamethoxazole; NAL, nalidixic acid; CIP, ciprofloxacin; OFX, ofloxacin.

b Boldface numbers represent the isolates that are resistant to the tested antimicrobial agents.

**FIG 2:**
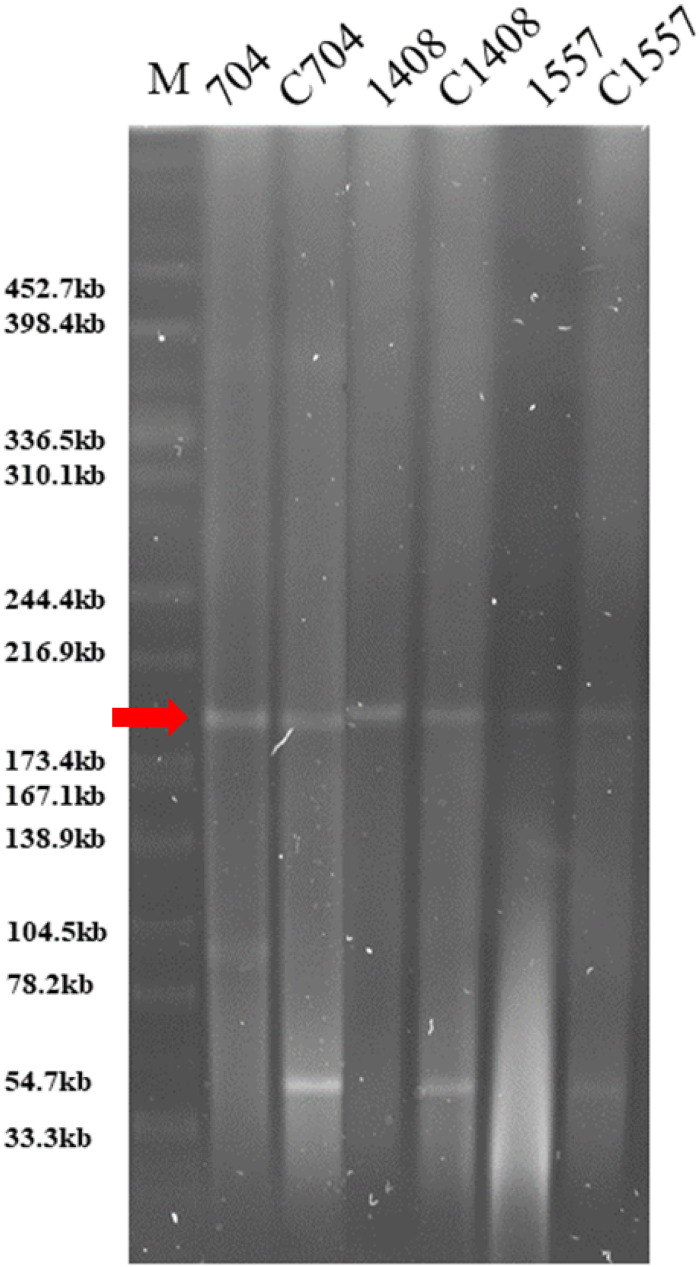
S1-PFGE profiles of the donor strains (*Vibrio* species strains 704, 1408, and 1557) and the corresponding transconjugants (C704, C1408, and C1557). Lane M: XbaI-digested genomic DNA fragments of Salmonella enterica serotype Braenderup H9812 were included as the size marker. The red arrow indicates the bands of conjugative plasmid.

To investigate the transmissibility of the *qnrS* gene and the genetic contexts, all 34 *qnrS*-bearing strains were subjected to conjugation assays. The *qnrS2* gene from 11 isolates and the corresponding resistance phenotype were successfully transferred to Escherichia coli J53, suggesting that the 11 isolates harbored the *qnrS2*-positive genetic structures in conjugative plasmids or other mobilizable genetic elements. Pulsed-field gel electrophoresis with S1 nuclease (S1-PFGE) revealed that the *qnrS2* gene in both parental strains and transconjugants was harbored by a plasmid of approximately 190 kb (Fig. 2). The MIC values of ciprofloxacin in the transconjugants were enhanced significantly compared with the recipient, rising from 0.015 to 1~4 μg/mL (Table 2). In addition, the levels of resistance to ampicillin, cefotaxime, tetracycline, chloramphenicol, and trimethoprim-sulfamethoxazole were also found to have increased significantly in the transconjugants.

**TABLE 2:** Antimicrobial susceptibility profiles of conjugative *qnrS2*-carrying *Vibrio* strains and the corresponding transconjugants

Strain ID	Species	MIC (μg/mL)^*a*^^,^^*b*^												
		AMP	CRO	CTX	MEM	AMC	AMK	GEN	TET	CHL	SXT	NAL	CIP	OFX
301	V. alginolyticus	**>64**	0.12	0.06	0.015	8	1	1	**16**	**32**	**>8**	8	1	1
C301	E. coli J53	**32**	0.06	0.06	006	4	2	0.5	**>32**	**>32**	**>8**	4	**1**	0.5
661	V. alginolyticus	**>64**	**>16**	**>16**	0.06	**32**	2	1	**16**	**32**	**>8**	8	2	1
C661	E. coli J53	8	0.06	0.06	0.015	2	1	0.06	**>32**	**>32**	**>8**	16	**4**	1
704	V. alginolyticus	**>64**	**>16**	**>16**	0.12	8	2	1	**32**	16	**>8**	4	**4**	1
C704	E. coli J53	**>64**	**4**	**8**	0.06	**>64**	1	0.5	**>32**	**>32**	**>8**	16	**4**	1
945	V. alginolyticus	**>64**	**>16**	**>16**	0.06	**32**	4	1	**16**	16	**>8**	4	2	0.5
C945	E. coli J53	**>64**	**4**	**8**	0.03	**>64**	2	0.5	**>32**	**>32**	**>8**	16	**4**	1
957	V. Parahaemolyticus	**>64**	**>16**	**>16**	0.0075	16	8	**32**	0.5	4	**>8**	8	0.5	1
C957	E. coli J53	**>64**	0.06	0.06	0.06	**>64**	2	0.5	8	**>32**	**>8**	**32**	2	2
1408	V. alginolyticus	**>64**	**>16**	**>16**	0.06	4	2	2	**>32**	1	0.25	8	2	1
C1408	E. coli J53	**>64**	**4**	**8**	**4**	**>64**	0.5	0.5	**>32**	**>32**	2	**32**	**4**	2
1557	V. alginolyticus	**>64**	**>16**	**>16**	0.06	**32**	4	1	**32**	8	**>8**	8	**4**	2
C1557	E. coli J53	16	0.06	0.06	0.03	4	0.5	0.25	**>32**	4	**>8**	16	**4**	4
1942	V. alginolyticus	**>64**	**>16**	**16**	0.06	16	8	2	**32**	16	**>8**	2	1	0.5
C1942	E. coli J53	**64**	**4**	**4**	0.12	**>64**	0.5	1	**>32**	**>32**	**>8**	16	2	1
1974	V. alginolyticus	**>64**	**>16**	**>16**	0.015	16	2	0.5	**32**	**>32**	**>8**	2	2	2
C1974	E. coli J53	8	0.06	0.06	0.03	4	1	0.5	**>32**	4	**>8**	16	2	1
2089	V. alginolyticus	**>64**	**>16**	**>16**	0.12	**64**	4	1	**32**	**32**	**8**	2	1	0.25
C2089	E. coli J53	**64**	**4**	**8**	**4**	**>64**	1	0.5	**>32**	**>32**	**>8**	**32**	**4**	2
2111	V. Parahaemolyticus	**>64**	**>16**	**>16**	0.25	16	4	2	**32**	**>32**	**8**	4	1	0.5
C2111	E. coli J53	**>64**	**4**	**4**	0.25	**>64**	1	0.12	**>32**	**>32**	**>8**	16	2	1
J53^AZR^	E. coli	2	0.06	0.06	0.015	4	1	0.25	0.5	4	0.25	2	0.015	0.015

a AMP, ampicillin; CRO, ceftriaxone; CTX, cefotaxime; MEM, meropenem; AMC, amoxicillin-clavulanic acid; AMK, amikacin; GEN, gentamicin; TET, tetracycline; CHL, chloramphenicol; SXT, trimethoprim-sulfamethoxazole; NAL, nalidixic acid; CIP, ciprofloxacin; OFX, ofloxacin.

b Boldface numbers represent the isolates that are resistant to the tested antimicrobial agents.

**FIG 3:**
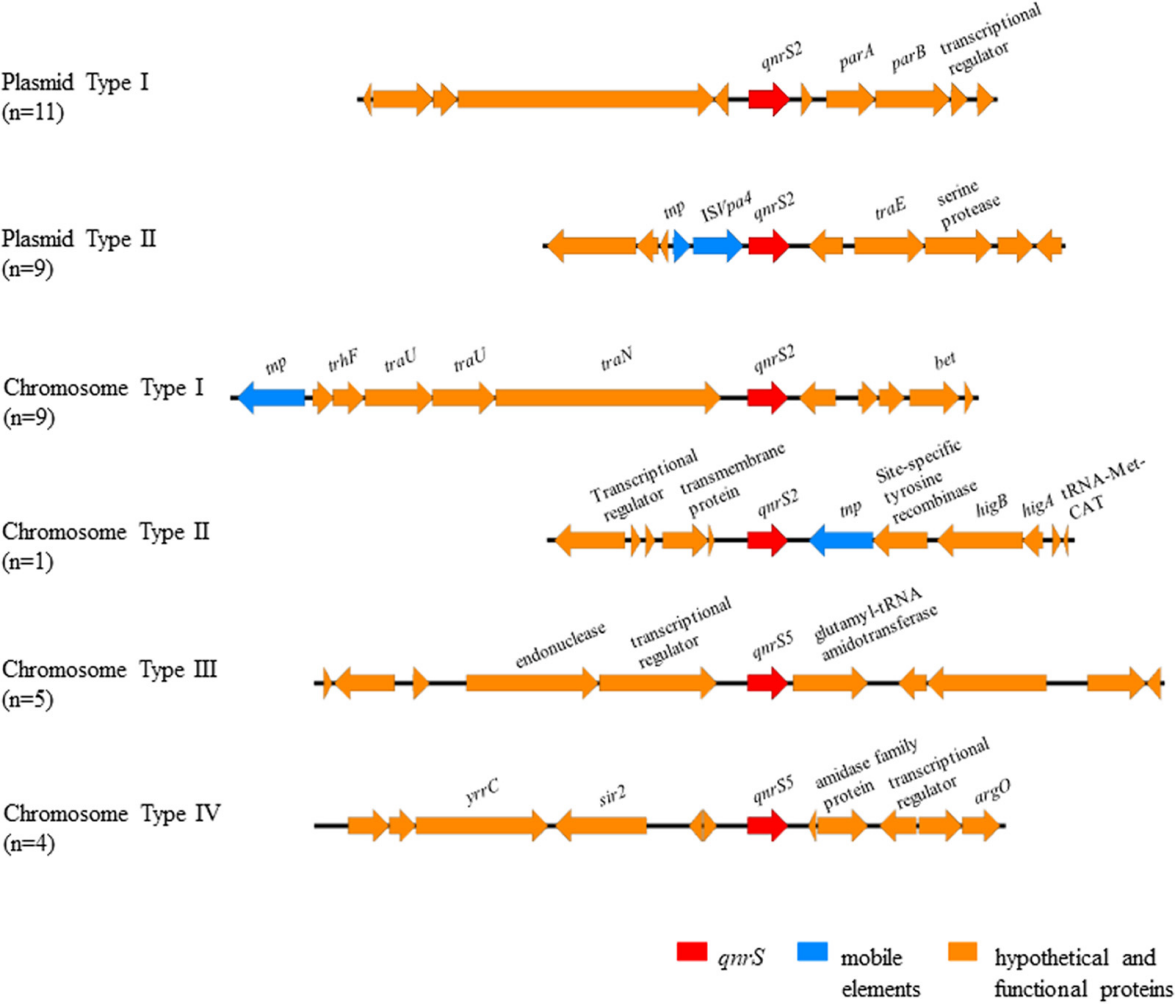
Schematic representation of different genetic structures surrounding the *qnrS* gene in *Vibrio* strains. The nucleotide sequences of the *qnrS2*-bearing region in plasmids pC704 and p1762 were selected as the representatives of plasmid types I and II, respectively. Selected sequences of strain 839 and strain 2129 were used to elucidate the genetic environment of chromosomal *qnrS2* types I and II. Selected sequences of strain 2175 and strain 818 were used to elucidate the genetic environment of chromosomal *qnrS5* types III and IV. Predicted genes are shown as arrows, and their orientation of transcription is indicated by arrowheads. The arrow size is proportional to the length of predicted genes. Predicted genes with different functions are colored as follows: red, *qnrS* genes; blue, mobile element genes; orange, genes encoding hypothetical proteins and other functional proteins.

Genome sequencing analysis of *qnrS*-carrying isolates identified in this study showed that the *qnrS2* genes could be located in the chromosome or the plasmids, whereas all the *qnrS5* genes recovered from this study were located in the chromosome (Fig. 1). The genetic environment of the plasmid-borne *qnrS2* gene could be categorized into two distinct groups (plasmid types I and II), involving one conjugative plasmid and one nonconjugative plasmid. Ten isolates carrying the chromosomal *qnrS2* gene exhibited two distinct gene environments, nine of which were surrounded by genes encoding conjugal transfer proteins (chromosome type I). Only one strain, 2129, was flanked by genes encoding the transposase-related proteins and the transmembrane protein (chromosome type II). The genetic context of *qnrS5*-harboring isolates was divided into two types (chromosome types III and IV). However, none of the *qnrS5* genes were surrounded by transposons, which differed from the *qnrS2* genes (Fig. 3). Phylogenetic analysis showed that *Vibrio* species strains carrying similar *qnrS*-carrying mobile elements tended to cluster together, but they were not clonally related (Fig. 1).

**FIG 4:**
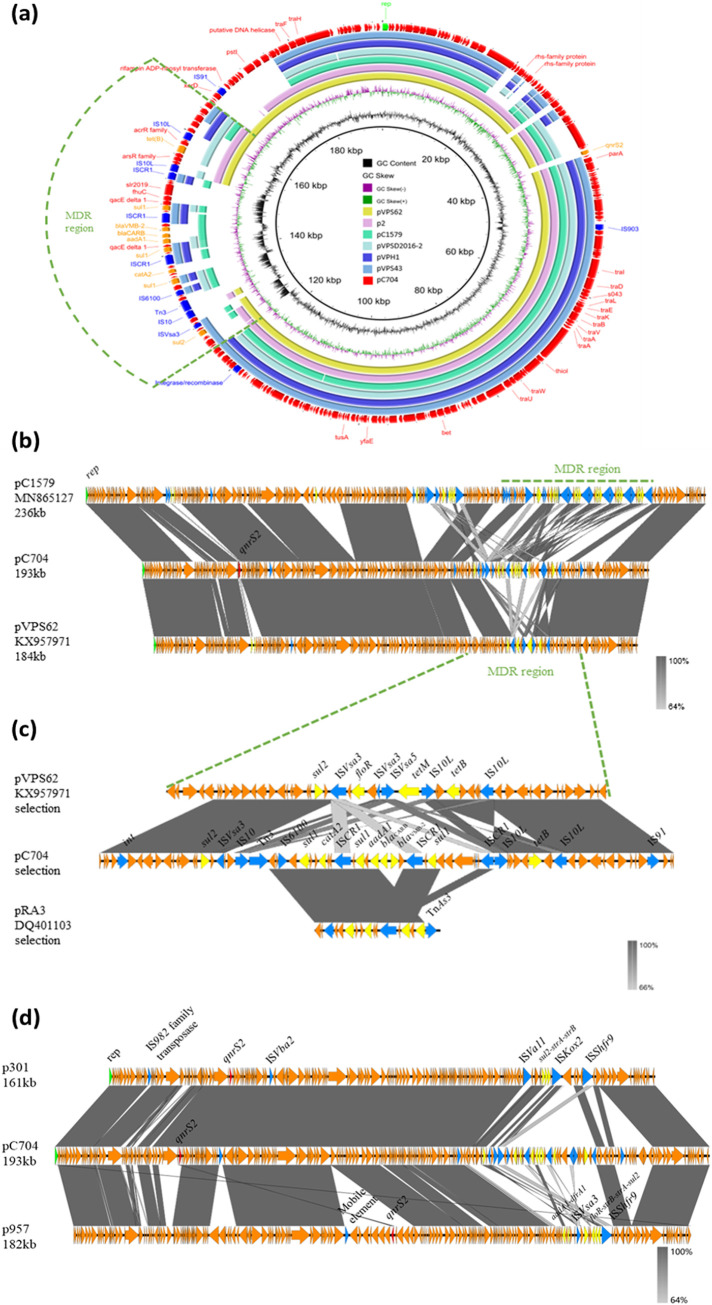
Comparison of the genetic characteristics of the pAQU-like plasmid carrying *qnrS2* recovered in this study and structurally similar plasmids using BRIG and Easyfig. (a) The complete sequence of pC704 (the outer circle) was used as a reference plasmid. The circular maps of the plasmids were generated using BRIG software and are depicted in the following order (inner to outer circles): pVPS62 (KX957971.1), p2 (CP030801.1), pC1579 (MN865127.1), pVPSD2016-2 (CP034301.1), pVPH1 (KP688397.1), pVPS43 (KX957970.1), and pC704. (b) Linear alignment of plasmid pC704 with two structurally similar plasmids pC1579 and pVPS62. (c) Linear alignment of the MDR region of pC704, pVPS62, and plasmid pRA3 (DQ401103.1). (d) Linear alignment of plasmid pC704 with two structurally similar *qnrS2*-bearing plasmids detected in this study, namely, p301 and p957.

Plasmids were extracted from six of the total transconjugants (C661, C704, C1408, C1557, C2089, and C2111) and were completely sequenced with the long-read Nanopore MinION platform. Sequence analysis showed that the *qnrS2* gene was located in the same plasmid in these six strains. The plasmid isolated from strain C704 was selected as the representative for investigation of the genetic features of the *qnrS2*-bearing conjugative plasmids. This conjugative *qnrS2*-bearing plasmid was 193,433 bp in length, contained 228 predicted coding sequences (CDSs), and exhibited a GC content of 44.20%. This plasmid harbored different genes, including resistance genes, mobile genetic elements, genes that encode conjugative transfer functions, and hypothetical protein-coding genes. Annotation results showed that *qnrS2* was surrounded by hypothetical protein-coding genes and genes that encode chromosome-partitioning proteins ParA and ParB, and an IS*903* family transposase gene was located downstream. After comparison with the plasmid replicon database, the predicted replication initiation gene *rep* in pC704 could not be classified into any of the known incompatibility groups by PlasmidFinder. A blastn search showed that the plasmid pC704 was highly similar (≥75% coverage and >99.8% identity) to pAQU-type plasmids recorded in the NCBI database, including plasmid pVPS62 (GenBank ID KX957971.1), p2 (CP030801.1), pC1579 (MN865127.1), pVPSD2016-2 (CP034301.1), pVPH1 (KP688397.1), and pVPS43 (KX957970.1). Comparison of the circular and linear images of pC704 with other similar pAQU-type plasmids showed that genetic variations were mainly located in the MDR region (Fig. 4a and b). The MDR region of pC704 was found to contain the sulfonamide resistance genes (*sul1* and *sul2*), chloramphenicol resistance gene (*catA2*), aminoglycoside resistance gene (*aadA1*), β-lactam resistance genes (*bla*_CARB-12_ and *bla*_VMB-2_), and the tetracycline resistance gene (*tetB*); these genes were surrounded by various insertion sequences and the Tn*3* transposon. It should be noted that an MDR-coding cluster, *sul1-aadA1-bla*_CARB-12_*-bla*_VMB-2_, was flanked by two copies of IS*CR1*. A tetracycline resistance gene *tetB* was flanked by two copies of IS*10L*. This MDR region exhibited a high degree of similarity to that of a plasmid, pRA3, which was isolated from Aeromonas hydrophila. However, two β-lactam resistance genes (*bla*_CARB-12_ and *bla*_VMB-2_) harbored by pC704 were absent in plasmid pRA3 (Fig. 4c). Strikingly, the *qnrS2* resistance gene harbored by pC704 was absent in other pAQU-type plasmids. Further sequence comparative analysis of other *qnrS2*-carrying isolates revealed that 11 conjugative isolates possessed a structurally similar pAQU-type plasmid backbone and could be grouped into plasmid type I. Two *qnrS2*-carrying isolates, strains 301 and 957 (the plasmids of which were designated p301 and p957, respectively), which were also included in this group, were found to possess a plasmid with a similar backbone but structurally different MDR region (Fig. 4d). To be more specific, instead of carrying an IS*6100*-*sul1*-*catA2*-IS*CR1*-*sul1*-*aadA1*-*bla*_CARB-12_-*bla*_VMB-2_-IS*CR1* MDR cluster in pC704, there was a *sul2*-*strA*-*strB* MDR-coding gene cassette flanked by IS*Va11* and IS*Kox2* in plasmid p301 and a *floR*-*strB*-*strA*-*sul2* MDR-coding cluster surrounded by IS*Vsa3* and IS*Shfr9* in plasmid p957.

**FIG 5:**
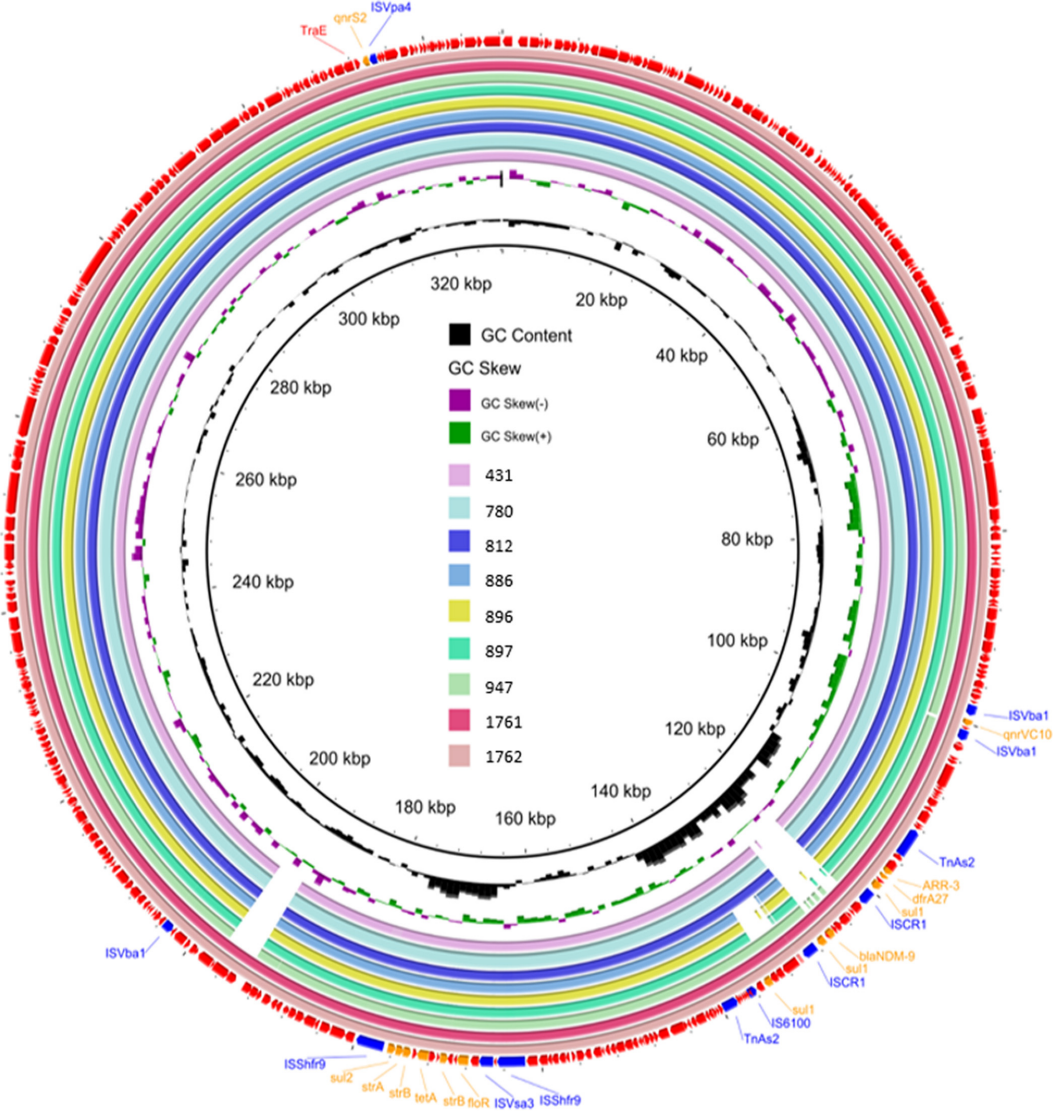
Genetic characteristics of the nonconjugative plasmids carrying *qnrS2*. The plasmid pVb1762 (accession number OK146920) was used as a reference to compare with other structurally similar plasmids.

The gene context of the nontransferable *qnrS2* gene found in this study was highly similar to that of a novel plasmid, pVb1762 (accession number OK146920), which was reported by our laboratory previously (Fig. 5) and was designated plasmid type II. In this novel plasmid, *qnrS2* was flanked by IS*Vpa4* and genes encoding hypothetical proteins. An MDR region within an 18,115-bp composite transposon, IS*Shfr9*-IS*Vsa3*-*floR*-*strB*-*tetA*-*strB*-*strA*-*sul2*-IS*Shfr9*, was located in this plasmid. Upstream of this transposon structure was an MDR cassette in which various resistance genes were flanked by the Tn*As2* and IS*CR1* elements. Another PMQR determinant, *qnrVC10*, which was accompanied by two IS*Vba1*, was also located in this plasmid. Conjugation experiments showed that this *qnrS2*-bearing plasmid was not transferrable to the recipient strain.

## DISCUSSION

*Vibrio* species are bacterial pathogens that may cause food contamination at various stages of food processing from production to consumption. In our previous study, we reported the screening of *qnrVC* genes in a total of 1,811 *Vibrio* species strains isolated from 2,051 food samples (including pork, beef, chicken, and shrimp) purchased in Shenzhen, Guangdong province, China, during the period from August 2015 to April 2017 (20). In this study, we conducted a comprehensive characterization of *Vibrio* species strains carrying *qnrS*, the gene commonly reported to mediate expression of phenotypic ciprofloxacin resistance in other Gram-negative pathogens such as Salmonella (19, 21). To date, *qnrS* is mainly detected in *Aeromonas* spp. and Enterobacter spp. but rarely in isolates of Klebsiella pneumoniae, *Pseudoalteromonas*, Pseudomonas, Shigella flexneri, and *Shewanella*. Reports of detection of *qnrS* in *Vibrio* spp. are also rare.

Overuse and abused usage of fluoroquinolones in aquaculture and health care facilities in the past decade resulted in a marked increase in the prevalence of quinolone resistance (22). Acquisition of the ability to express quinolone resistance is principally associated with mutational changes in the QRDR of the *gyrA* and *parC* genes. However, recent studies showed that PMQR genes are able to confer low levels of quinolone resistance and complement other chromosomal mechanisms, resulting in higher levels of resistance. Consistently, data from our study showed that carriage of *qnrS* or other PMQR genes, such as *qnrVC* and *qnrA*, without mutations in the QRDR in *Vibrio* spp. enabled the MIC range toward ciprofloxacin to vary from 0.25 mg/mL to 4 mg/mL. Such genes were also detectable among the *qnrS*-positive *Vibrio* isolates. Eleven of the total *qnrS*-bearing isolates were found to harbor point mutations in the QRDRs, with the Ser83Ile substitution in GyrA being the most frequently detected amino acid substitution. Isolates with a single QRDR mutation exhibited resistance toward ciprofloxacin, with MIC values ranging from 4 mg/mL to 16 mg/mL, whereas QRDR mutation together with single or multiple PMQR genes exhibited high-level resistance. It is noticeable that the QRDR mutation sites in V. parahaemolyticus and V. alginolyticus strains were different. It appeared that mutations in both the *gyrA* and *parC* genes occurred only in V. alginolyticus isolates, whereas the V. parahaemolyticus isolates only possessed a single mutation at codon 83 of the *gyrA* gene or codon 85 of the *parC* gene. However, several reports indicated that point mutations in *gyrA* at position 83 (Ser→Ile) and *parC* at position 85 (Ser→Leu) were also detected in strains of V. parahaemolyticus isolated from food samples (23, 24). This discrepancy between V. parahaemolyticus and V. alginolyticus might be the result of limited numbers of strains included in this study. These findings suggest that PMQR genes contributed significantly to the development of ciprofloxacin resistance in *Vibrio* spp.

Our study showed that the *qnrS5* gene was frequently located in the chromosome of *Vibrio* species strains and that *qnrS2* could be found in both the chromosome and plasmids. The *qnrS2* allele has been commonly found in IncQ, IncU, and ColE-type plasmids. In these plasmids, *qnrS2* is part of a mobile insertion cassette, which lacks the transposase gene but is flanked by 22-bp imperfect inverted repeats and 5-bp direct repeats. Recently, a new surrounding genetic structure has been described for the *qnrS2* gene, in which *qnrS2* was flanked by two IS*26* elements (5). However, sequence analysis of *qnrS2*-carrying isolates in this study did not observe the gene environment described above, indicating that a distinct genetic context was involved in dissemination of the *qnrS2* gene among *Vibrio* spp. The genetic environment of the plasmid-borne *qnrS2* genes identified in this study could be divided into two distinct groups (plasmid types I and II), which involve one conjugative plasmid and one nonconjugative plasmid. The *qnrS2*-harboring conjugative plasmid has a backbone similar to that of the pAQU-type plasmids but contains different resistance genes. The pAQU-type plasmids, namely, pAQU1 and pAQU2, were first identified in marine pathogens in Japan (23, 24) and were subsequently disseminated as MDR conjugative plasmids to important bacterial pathogens in Asia. Bacterial strains harboring the pAQU group plasmids mainly belong to *Vibrio* spp. and Photobacterium damselae subsp. *damselae*, suggesting that such plasmids are mainly maintained and disseminated in the aquatic environment and are therefore responsible for the dissemination of antibiotic resistance genes (ARGs) among marine bacteria (25). It was speculated that integration of various mobile elements, such as IS*10*, IS*CR1*, IS*91*, and IS*6100*, into the plasmid backbone was the key mechanism by which exogenous resistance genes were acquired during the evolution process (24). However, the *qnrS2* gene observed in our study was surrounded by a variety of hypothetical protein-coding genes. The functions of these hypothetical proteins need to be further investigated. Conjugation experiments in this study demonstrated that the *qnrS2*-carrying pAQU-type plasmids could be transferred from *Vibrio* spp. to E. coli J53 and caused a reduction in the susceptibility of E. coli J53 to fluoroquinolones. Moreover, the MIC value of two cephalosporins, cefotaxime and ceftriaxone, in some of the transconjugants also increased from 0.06 μg/mL to 1~4 μg/mL. The MIC data showed that most of these isolates were able to transfer the conjugative plasmid that encodes coresistance to two clinically important antibiotics, ciprofloxacin and cephalosporins, to the recipient strain. The unique features observable in the genetic environments of the *qnrS2* gene carried by foodborne *Vibrio* isolates in this study and those of the previously reported *qnrS2*-carrying strains indicated that active evolution and dissemination of *qnrS2* occur among strains of *Vibrio* spp. Our findings therefore suggest that foodborne bacteria, such as those of the *Vibrio* spp., constitute a key reservoir of resistance genes, which may be transmissible to other human pathogens during food production and processing.

## MATERIALS AND METHODS

### Screening of the *qnrS* gene.

Food-borne *Vibrio* isolates were collected from wet markets and supermarkets in Shenzhen, Guangdong province, China, during the period between August 2015 and April 2017, as reported in our former study (20). Bacterial DNA was extracted using the rapid boiling method (26); the *qnrS* gene was screened by a previously described PCR method (27). The *qnrS*-positive isolates were then subjected to molecular screening for *qnrA*, *qnrB*, *qnrC*, *qnrD*, *qnrVC*, *aac(6′)lb-cr*, and *oqxAB*, as previously described (28). Subsequently, *qnrS*-bearing isolates were subjected to antimicrobial susceptibility tests following the standard agar dilution method as described by the Clinical and Laboratory Standards Institute (29). Escherichia coli strain ATCC 25922 and Staphylococcus aureus strain ATCC 29213 were used as quality control strains. The resistance breakpoints published by the Clinical and Laboratory Standards Institute were used in the tests (30). All 34 *qnrS*-carrying isolates were sequenced by the Illumina platform and subjected to further analysis. Subsequently, MLST analysis was performed by following the guidelines of PubMLST (https://pubmlst.org/organisms/vibriospp).

### Filter mating assay and S1-PFGE.

Conjugation assays were performed to evaluate the transferability of *qnrS*-carrying genetic elements. E. coli strain J53 AZR was used as the recipient strain. The donor and recipient strains were grown at a log phase in LB broth and mixed at a donor:recipient ratio of 1:3 and applied to a 0.22-μm filter, followed by culturing at 37°C for 16 h. Transconjugants were selected on eosin methylene blue (EMB) agar plates containing 0.5 μg/mL ciprofloxacin and 100 μg/mL sodium azide. The genetic identity of transconjugants harboring *qnrS* was confirmed by PCR, and the MIC values of various antibiotics were determined for the transconjugants as described above. To investigate the *qnrS*-bearing plasmid profiles of *qnrS*-positive strains and their transconjugants, total DNA was digested with S1 nuclease, followed by PFGE with the CHEF MAPPER system (Bio-Rad, CA, USA) (31).

### Plasmid sequencing and bioinformatic analysis.

Plasmids were collected from the test strains by using the Qiagen plasmid midi kit (Qiagen, Valencia, CA). The Illumina platform and the Nanopore MinION long-read sequencing platform were used to generate the draft whole-plasmid maps. The Illumina paired-end libraries were prepared by using the NEBNext Ultra DNA library prep kit for Illumina (New England Biolabs) and sequenced on an Illumina NextSeq 500 platform. The library for MinION sequencing was prepared using the Oxford Nanopore Technologies (ONT) sequencing kit (rapid barcoding kit 96; SQK-RBK110.96). *De novo* assemblies of MinION long reads and Illumina reads were generated by using SPAdes 3.12.1 (32) and the CLC Genomics Workbench (CLC bio, Denmark), respectively. Long assembled contigs obtained from MinION long reads were used to align and join the contigs obtained from the Illumina assembly results. The completed plasmid sequence was annotated by the RAST tool (33) and the NCBI Prokaryotic Genome Annotation Pipeline (PGAP). The phylogenetic tree of these *Vibrio* species strains was constructed using Roary (34) and FastTree (35) based on the single-nucleotide polymorphisms (SNPs) of the core genomes. BRIG (36) and Easyfig (37) were used to generate multiple and pairwise sequences for comparison of the plasmids tested in this study.

### Data availability.

The plasmid sequence of the pAQU-type conjugative plasmid pC704 found in this study was submitted to the NCBI database with accession number OP958859.
